# Brain monitoring using near-infrared spectroscopy to predict outcome after cardiac arrest: a novel phenotype in a rat model of cardiac arrest

**DOI:** 10.1186/s40560-020-00521-9

**Published:** 2021-01-07

**Authors:** Ryosuke Takegawa, Kei Hayashida, Rishabh Choudhary, Daniel M. Rolston, Lance B. Becker

**Affiliations:** 1 Laboratory for Critical Care Physiology, Feinstein Institutes for Medical Research, Northwell Health System, Manhasset, NY USA; 2 Department of Emergency Medicine, North Shore University Hospital, Northwell Health System, Manhasset, NY USA

**Keywords:** Cardiac arrest, Cardiopulmonary resuscitation, Near-infrared spectroscopy, NIRS, Cerebral oxygen saturation, rSO_2_, Neurological outcome, Prognostication, Cerebral oximetry index, COx

## Abstract

Improving neurological outcomes after cardiac arrest (CA) is the most important patient-oriented outcome for CA research. Near-infrared spectroscopy (NIRS) enables a non-invasive, real-time measurement of regional cerebral oxygen saturation. Here, we demonstrate a novel, non-invasive measurement using NIRS, termed modified cerebral oximetry index (mCOx), to distinguish the severity of brain injury after CA. We aimed to test the feasibility of this method to predict neurological outcome after asphyxial CA in rats. Our results suggest that mCOx is feasible shortly after resuscitation and can provide a surrogate measure for the severity of brain injury in a rat asphyxia CA model.

Letter to the Editor

Improving neurological outcomes after cardiac arrest (CA) is likely the most important patient-oriented outcome for CA research. Invasive brain monitoring of intracranial pressure or cerebral oxygenation is rarely used in patients with CA due to the risk of bleeding and infectious complications. Near-infrared spectroscopy (NIRS) enables a non-invasive, real-time measurement of regional cerebral oxygen saturation (rSO_2_). Recent research has demonstrated a correlation between dynamic changes in mean arterial pressure (MAP) and concurrent changes in rSO_2_, suggesting NIRS has the potential to assess cerebral vasoreactivity [1, 2]. It is based on assumption that changes in rSO_2_ are proportional to changes in cerebral blood flow over brief periods with stable cerebral metabolic rate. Other studies have shown that a moving correlation coefficient between MAP and rSO_2_ (a value between − 1 and + 1), termed the cerebral oximetry index (COx), was an independent predictor of outcomes in patients with CA [3, 4]. A positive COx suggests impaired autoregulatory vasoreactivity (in most studies at a threshold of > 0.3), whereas a negative or near zero correlation indicates intact vasoreactivity. In most studies, COx is determined by using ICM+ software (University of Cambridge, Cambridge, UK). Although real-time, non-invasive brain monitoring plays a major role in the management of post-CA syndrome, there are no studies examining whether brain monitoring early after the return of spontaneous circulation (ROSC) enables the prognostication of neurological recovery. Establishing reliable surrogates to assess neurological recovery, which can be obtained immediately after ROSC, is an unmet medical need for developing novel strategies to improve outcomes after CA. By using a rat asphyxial CA model, we developed a modified COx (mCOx) which can be calculated without the specific ICM+ software. We aimed to test the feasibility of mCOx measured shortly after ROSC to assess neurological recovery.

TOS-QQ® (TOSTEC, Co., Tokyo, Japan) measures rSO_2_ every second based on the Beer-Lambert law using two different wavelengths (770 and 870 nm) of near-infrared LED light. After approval by the Institutional Animal Care and Use Committees (IACUC) at Feinstein Institutes for Medical Research, Sprague-Dawley male rats (400–500 g) were subjected to 6 min or 12 min of vecuronium (2 mg/kg) induced-asphyxial CA followed by cardiopulmonary resuscitation (CPR) with finger chest compressions and a bolus of epinephrine (20 μg/kg). TOS-QQ® was attached on hair-shaved head of rats. All resuscitated rats inhaled 100% O_2_ via mechanical ventilator during rSO_2_ measurement. The esophageal temperature was maintained at 37 ± 0.5 °C and end-tidal CO2 was controlled at 40 ± 5 mmHg until the end of the experiment. Neurologic function score at 24 h after ROSC was assessed by an investigator blinded to the experiment (0–100 scale; 0 = brain death, 100 = normal). We also monitored the brain electrical activity by electroencephalogram (EEG) after ROSC. Small holes were created in the skull and bilateral stainless epidural electrodes were placed over the frontal and parietal area. To measure mCOX, digital NIRS (a number that includes one decimal place) and MAP (an integer number) signals were synchronously recorded every 1 s. Data were directly collected to a computer and analyzed by Microsoft Excel® as follows: rSO_2_ and MAP signals were used for a 180-s time window to calculate the Spearman correlation coefficient, which were updated every 90 s. The mCOx was defined as a mean of 5 sequential coefficients (6-min time window) prior to the time of measurement. Given epinephrine administration affects cerebral oxygenation and metabolism immediately after CPR [5], rSO_2_ measurements were started at 15 min (i.e., mCOx recording was started at 21 min post-ROSC). Then, mCOx measurements were continued for 30 min.

Neurofunction score was better in rats subjected to 6 min CA compared to 12 min CA (Fig. 1a). In terms of EEG, rats subjected to 12 min CA have a markedly longer duration from ROSC to the onset of both appearance of EEG amplitude activity (> 5% of the basal value with no EEG activity) and continuous EEG, confirming that 12 min CA induced more severe brain damage than 6 min CA (Fig. 1b). The rats subjected to 12 min CA had markedly higher mCOx values early after CA compared to rats subjected to 6 min CA (Fig. 1c).

**Fig. 1:**
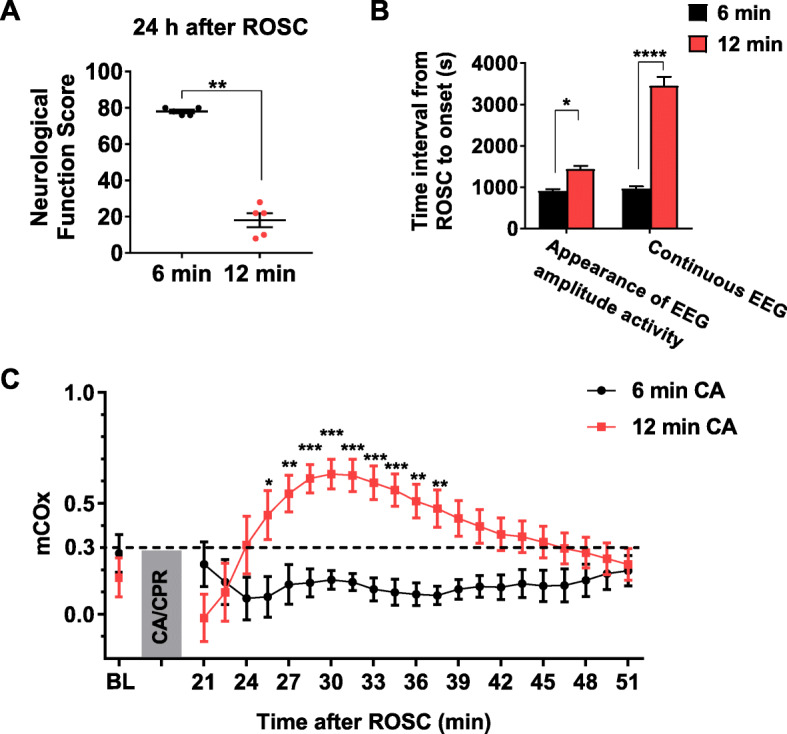
**a** Neurological functional scores 24 h after cardiac arrest (CA) in rats subjected to 6 min CA and 12 min CA. Mann-Whitney *U* test was used to test statistical difference. *n* = 5 for each group. **b** Brain electrical recovery after CA measured by electroencephalogram. Two-way ANOVA followed by Sidak’s corrections was used to test statistical difference. *n* = 3 for each group. **c** Serial modified Cox (mCOx) measurements early after successful resuscitation among groups. Two-way repeated measures ANOVA followed by Sidak’s corrections was used to test statistical difference. *n* = 12 for each group. Data are presented as mean ± SEM. *P < .05, **P < .01, ***P < .001, ****P < .0001

Our results suggest that mCOx measurement is feasible shortly after ROSC and can provide a surrogate measure for the severity of brain injury in rats. However, it should be noted that single measurement shortly after ROSC may not identify the time-varying aspects of the different physiological mechanisms. The exact mechanism of transient increase of mCOX early after ROSC in the 12 min CA group remains unknown. However, given that the duration of increased mCOX was consistent with the phase with undetectable continuous EEG activity, it can be speculated that impaired neural activity and metabolism may affect cerebral vascular reactivity and autoregulation. Further studies are warranted to determine whether the use of mCOx monitoring and concomitant treatment strategies can improve post-CA outcomes.

## Data Availability

The datasets generated and/or analyzed during the current study are available from the corresponding author on reasonable request.

## Abbreviations

CA: Cardiac arrest
NIRS: Near-infrared spectroscopy
rSO_2_: Regional cerebral oxygen saturation
MAP: Mean arterial pressure
COx: Cerebral oximetry index
ROSC: Return of spontaneous circulation
mCOx: Modified cerebral oximetry index
CPR: Cardiopulmonary resuscitation
EEG: Electroencephalogram
